# AlMnPdPtAu Quasicrystal Modulated Carbon Nanotubes for H_2_ Sensors: Experimental and DFT Computational Analysis

**DOI:** 10.1021/acsami.5c00924

**Published:** 2025-04-01

**Authors:** Sumit Kumar, Anyesha Chakraborty, Juan Rafael Gomez Quispe, Rahul Mitra, Suraj Barala, Pedro Alves Da Silva Autreto, Chandra Sekhar Tiwary, Krishanu Biswas, Mahesh Kumar

**Affiliations:** † Department of Electrical Engineering, Indian Institute of Technology Jodhpur, Jodhpur 342030, India; ‡ School of Nano Science and Technology, Indian Institute of Technology, Kharagpur, West Bengal 721302, India; § Center for Natural and Human Sciences, Federal University of ABC, Santo André, SP 09280-560, Brazil; ∥ Department of Materials Science and Engineering, Indian Institute of Technology Kanpur, Kanpur 208016, India; ⊥ Department of Metallurgical and Materials Engineering, Indian Institute of Technology Kharagpur, Kharagpur 721302, India; # Inter-disciplinary Department of Space Science and Technology, Indian Institute of Technology Jodhpur, Jodhpur 342030, India; ∇ Department of Cybernetics, Nanotechnology and Data Processing, Faculty of Automatic Control, Electronics and Computer Science, Silesian University of Technology, Akademicka 16, Gliwice 44-100, Poland

**Keywords:** quasicrystal, H_2_ sensor, heterointerface, selectivity, dissociation, spillover effect

## Abstract

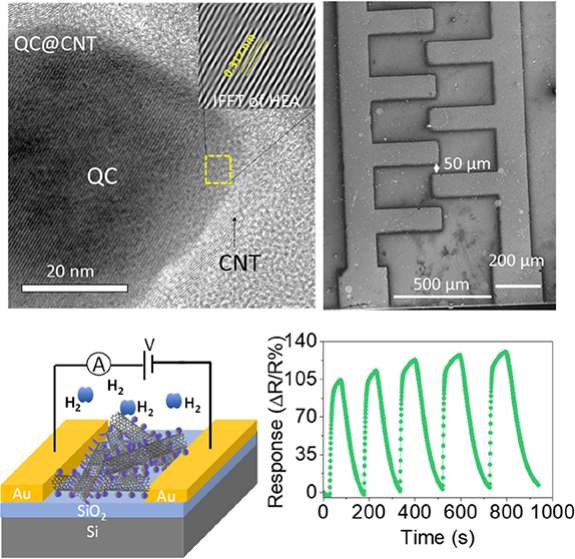

Hydrogen (H_2_) sensors are crucial for safety in H_2_ storage and fuel cell systems. AlMnPdPtAu high-entropy alloy (HEA) quasicrystal nanosheets with high surface area, tunable electronic structure, and atomic disorder exhibit enhanced H_2_ adsorption and sensor response. This work demonstrates highly sensitive and selective H_2_ sensors developed by a low-cost screen printing method using the AlMnPdPtAu quasicrystal and CNTs. The sensor demonstrates high sensitivity with a relative response of 103% (1 ppm) to 130.4% (100 ppm) and rapid dynamics (τ_res_ = 19 s, τ_rec_ = 81 s at 100 ppm of H_2_) at room temperature. Structural analysis by high-resolution transmission electron microscopy, X-ray diffraction, and Fourier transform infrared spectroscopy confirmed the interface formation between the AlMnPdPtAu HEA quasicrystal and CNTs. Raman spectroscopy revealed structural and electronic interactions within the composite, while X-ray photoelectron spectroscopy identified chemical states and surface interactions at the AlMnPdPtAu QC@CNT interface. The density functional theory study highlighted dissociative H_2_ adsorption modes and spillover effects at the QC@CNT interface, where H atoms bond with Mn atoms in the quasicrystal, facilitating H atom migration and stabilization. The adsorption energy and Bader charge transfer values are calculated to determine the binding strength of the H atom to the sensing material and the extent of electronic interaction, influencing sensitivity.

## 1 Introduction

To address climate change, energy systems are evolving to use technology that reduces greenhouse gas emissions.^1,2^ Hydrogen (H_2_) shows great potential as a renewable and clean energy carrier due to its environmentally friendly combustion properties resulting in the production of only H_2_O.^3,4^ Hydrogen is a highly combustible and explosive gas, which has a low ignition energy of ∼0.017 mJ. It has a wide flammability range in air, ranging from 4% to 75%. Developing susceptible, selective, and room-temperature-operated sensors is necessary for effectively detecting and monitoring H_2_ during production, transportation, storage, and final usage, ensuring long-term stability.^5^

Chemiresistive sensors, which detect changes in electrical resistance upon gas exposure, have emerged as promising tools for H_2_ monitoring due to their sensitivity and rapid response times. Recent advancements in nanomaterials, particularly metal oxides such as SnO_2_,^6^ WO_3_,^7^ ZnO,^8^ and TiO_2_,^9^ exhibit a strong sensitivity toward H_2_. These materials offer high surface area-to-volume ratios and tunable electronic properties, facilitating improved interaction with hydrogen molecules.^10,11^ However, their performance is restricted due to high operating temperatures and poor selectivity. Metals such as platinum (Pt) and palladium (Pd) have become increasingly popular in recent years as substitutes for metal-oxide-based H_2_ sensors. This is because H_2_ molecules dissociate on the surfaces of these metals, leading to significant changes in their resistances.^12,13^ It has been found that combining Pd with Pt metal significantly enhances the sensitivity of H_2_ detection, effectively doubling its performance.^14^ Other studies have shown that the addition of gold (Au) in the palladium–platinum (Pd–Pt) alloy improves the sensitivity and recovery time. Catalytically active multicomponent high-entropy alloy (HEA) solid components of five metal elements, such as Al, Cu, Ni, Pd–Pt, and Au, possess remarkable properties that allow for precise composition variations to achieve specific purposes. This tunability of composition allows the reduction or elimination of the need for highly valuable metals.^15,16^ HEAs can be fine-tuned to create a material system that provides exceptional selectivity and sensitivity for H_2_ adsorption using a resistance-driven sensor.

The “spinnable arrays” of CNTs exhibit impressive electrical and thermal conductivity and robust mechanical properties,^17^ and can be functionalized through covalent bonding.^18−26^ Additionally, they are essentially free from any residual catalyst materials.^27^ For sensor applications, the chemiresistor is a conducting element that experiences changes in electrical resistance when exposed to a specific gas. Researchers have chosen to utilize CNTs as a scaffold for enhancing their hydrogen-sensing capabilities by incorporating them with more effective materials like Pt and Pd.^28−31^

In the current work, we have synthesized a composition consisting of AlMnPdPtAu quasicrystal nanosheets of HEA and CNT. The quasicrystal nanosheets are utilized to make a composition with CNT. The hybrid composition is further used for gas-sensing properties. To gain insight into efficient H_2_ sensing, we performed calculations based on Density Functional Theory (DFT). It was found that there is a significant structural change at the interface of QC and CNT, due to charge transfer between the two components, which leads to its stability. The H_2_ adsorption was more favorable at HEA-QC sites rather than at CNT, which could explain the enhanced H_2_ gas sensing by the QC@CNT composite. The underlying mechanism for the enhanced sensing capabilities of the QC@CNT is corroborated by a comprehensive array of analyses of DFT calculations, revealing that the introduction of QC@CNT effectively enhances the spillover effect, adsorption, and surface area for H_2_ exposure, which achieves a profound modulation of the efficient adsorption and charge transfer, thus leading to the superior gas-sensing performance observed.

## 2 Experimental Section

### 2.1 Synthesis of the AlMnPdPtAu Quasicrystal (QC)

The high-purity elements Al (purity 99.95%, Alfa Aesar), Mn (purity 99.90%, Alfa Aesar), Pd (purity 99.99%, Alfa Aesar), Pt (purity 99.5%, Alfa Aesar), and Pd (purity 99.5%, Sigma-Aldrich) were used to synthesize the high-entropy alloy quasicrystal in a vacuum arc melting furnace. Al, Mn, Au, Pd, and Pt in an equimolar ratio (99.99% pure) were melted in an arc melting furnace to produce a metal ingot within an argon (Ar) environment to prevent oxidation of the metals. The ingot underwent several melting processes to make sure it was in a fully molten state. The as-cast button underwent homogenization at 1473 K within a vacuum-sealed quartz tube for 10 h and then underwent quenching in cold water to preserve the high-temperature single phase. Subsequently, the button underwent crushing into smaller pieces and was cryomilled in a liquid nitrogen environment at temperatures below 150 K for 6 h. This entire procedure is known as a casting-cum-comminution method, which proves to be highly effective in the production of nanocrystalline high-entropy alloys.^32,33^

### 2.2 Synthesis of Carbon Nanotubes (CNTs)

**Figure 1:**
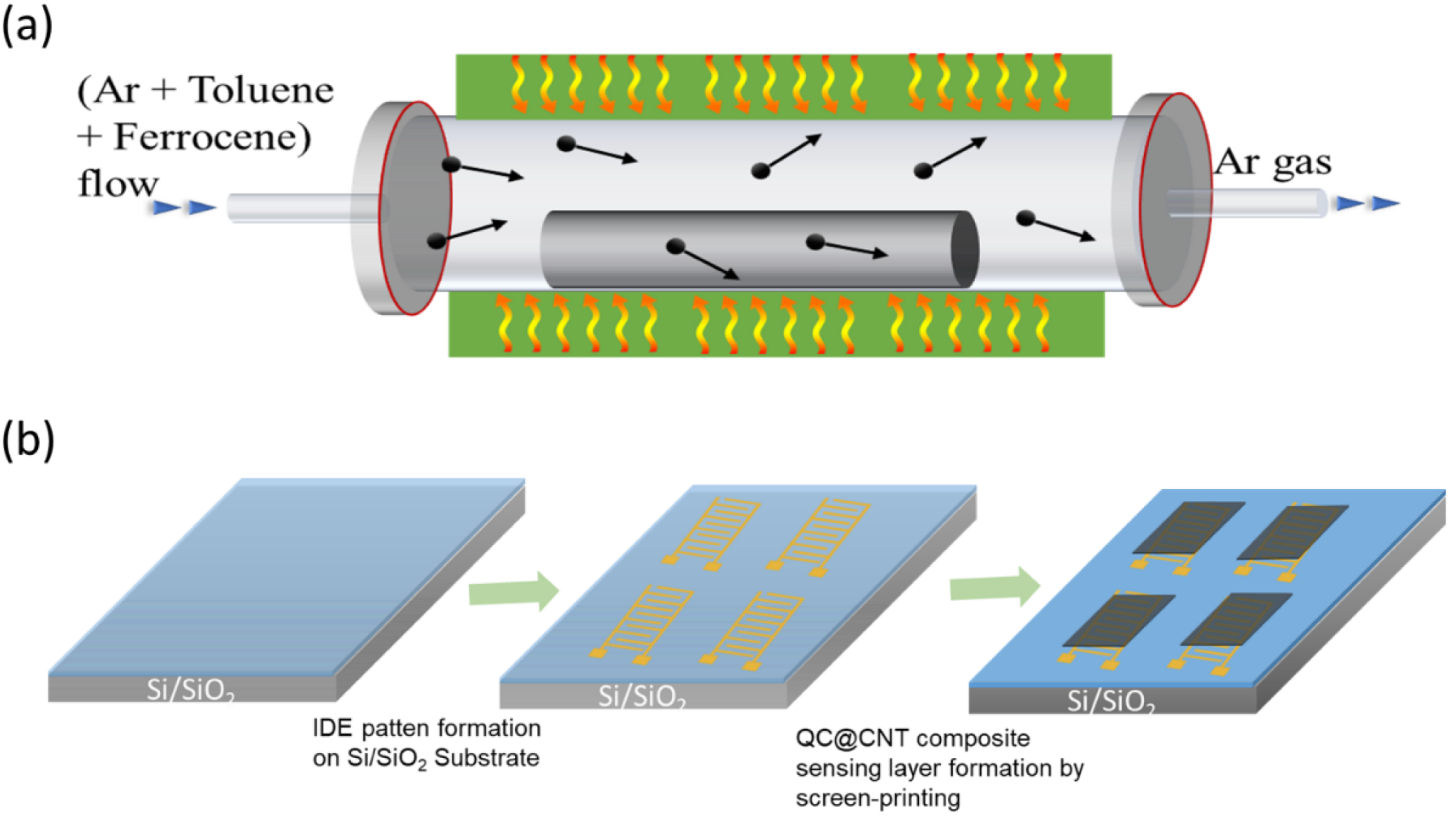
(a) Growth of carbon nanotubes by a conventional chemical vapor deposition system. (b) Schematic of QC@CNT sensor fabrication by the screen-printing technique on SiO_2_/Si substrates using Au/Cr IDE patterns.

Ferrocene and toluene precursors were utilized to synthesize carbon nanotubes (CNTs) through the conventional three-zone chemical vapor deposition (CVD) method within a tubular quartz tube (Figure 1a). Under thermal decomposition, Fe particles develop, which facilitate the breakdown of toluene and enhance the synthesis of nanotubes. A solution of 80 mg/mL ferrocene in toluene was prepared for the growth of CNTs. A total of 60 mL of this toluene and ferrocene catalyst solution was gradually purged into the CVD reactor at 700 °C for 10 min, utilizing a quartz tube and a flow of 120 sccm argon (Ar). This task was conducted within a quartz tube maintained at a pressure of 1 mbar. Following the reaction, the quartz tube was allowed to cool to room temperature. The dark reaction product was extracted from the small quartz tubes.^34,35^ The acid oxidation procedure was used to remove the metal particles. Approximately 100 mg of nanotubes were introduced into a 5 mL solution of boiling acid and refluxed for 60 min. After the sample was washed, it was carefully neutralized with deionized water until achieving a pH of 6. The CNTs were filtered and subsequently dried in an oven at approximately 100 °C in air.

### 2.3 Characterization

High-resolution transmission electron microscopy (HRTEM) (FEI Titan FEG, operating at 300 kV) was used to investigate the microstructural characteristics of the samples. The X-ray diffraction (XRD) patterns of CNT, QC, and QC@CNT were acquired using PANalytical X‘Pert PRO and Bruker D8 Advance diffractometers with Cu–Kα1 radiation. The chemical composition was determined using X-ray photoelectron spectroscopy (XPS) via a PHI VersaProbe III. The FTIR spectra were obtained using a model NICOLET 6700 instrument from Thermo Fisher Scientific.

### 2.4 Sensor Fabrication and Measurements

The fabrication process of the gas sensor is as follows: a SiO_2_/Si wafer was used to fabricate an interdigitated electrode (IDE) pattern of Au (250 nm)/Cr (20 nm) via the thermal evaporation technique (Figure 1b). The pad size of IDE was 200 × 200 μm^2^ with 50 μm finger spacing. A pure CNT, pure AlMnPdPtAu QC, an optimum amount of QC-decorated CNT, and an optimum amount of QC@CNT composite sensors were developed by using the screen-printing method. The precursors and parameters for sensor preparation are shown in Table 1. The composite films for the sensing layer were formed by adding specific quantities of CNT, a quasi-crystal, α-terpineol (binder), and ethylene glycol (binder). The variation in terpineol and ethylene glycol concentrations was optimized to ensure uniform dispersion, stability, and proper film formation for the CNT and composite materials. Different ratios prevented aggregation, improved adhesion, and enhanced the sensor performance. These components were mixed by using a magnetic stirrer to achieve a compositionally uniform paste for the defined samples (standard, modified, and reference samples). All sensors were fabricated by spreading the paste over an IDE-patterned SiO_2_/Si substrate, followed by the utilization of the screen-printing method to achieve a uniform sensing layer. Following the preparation of a uniform thin sensing layer, the device underwent treatment in a furnace at 100 °C for 1 h in an N_2_ atmosphere to enhance the adhesion and quality of the sensing layer.

**Table 1:** Sensor Types and Material in the Nanocomposites^a^

Sensors	MWCNTS	AlMnPdPtAu HEA QC	α-Terpineol	Ethylene Glycol
CNT	10 mg	0 mg	5 μL	2 μL
QC	0 mg	10 mg	3 μL	1 μL
QC@CNT	6 mg	4 mg	5 μL	2 μL

a QC-decorated CNT sensor developed via drop casting using a 10 μL droplet of 5 mg/1 mL suspension of AlMnPdPtAu HEA QC in Isopropyl Alcohol (IPA) on a screen-printed bare CNT sensor.

Gas-sensing tests were conducted using a probe station equipped with a gas inlet, and an outlet chamber, and the Keithley 2636B SourceMeter was used for precise measurement of current–voltage (*I*–*V*) and resistance variations in gas sensors by sourcing voltage and measuring current. It effectively monitors the sensor response. A constant bias voltage was applied to the two-terminal devices, monitoring real-time resistance signals. Gas concentrations were adjusted precisely in a mixing chamber and injected into the sensing chamber.

### 2.5 DFT Computational Details

**Figure 2:**
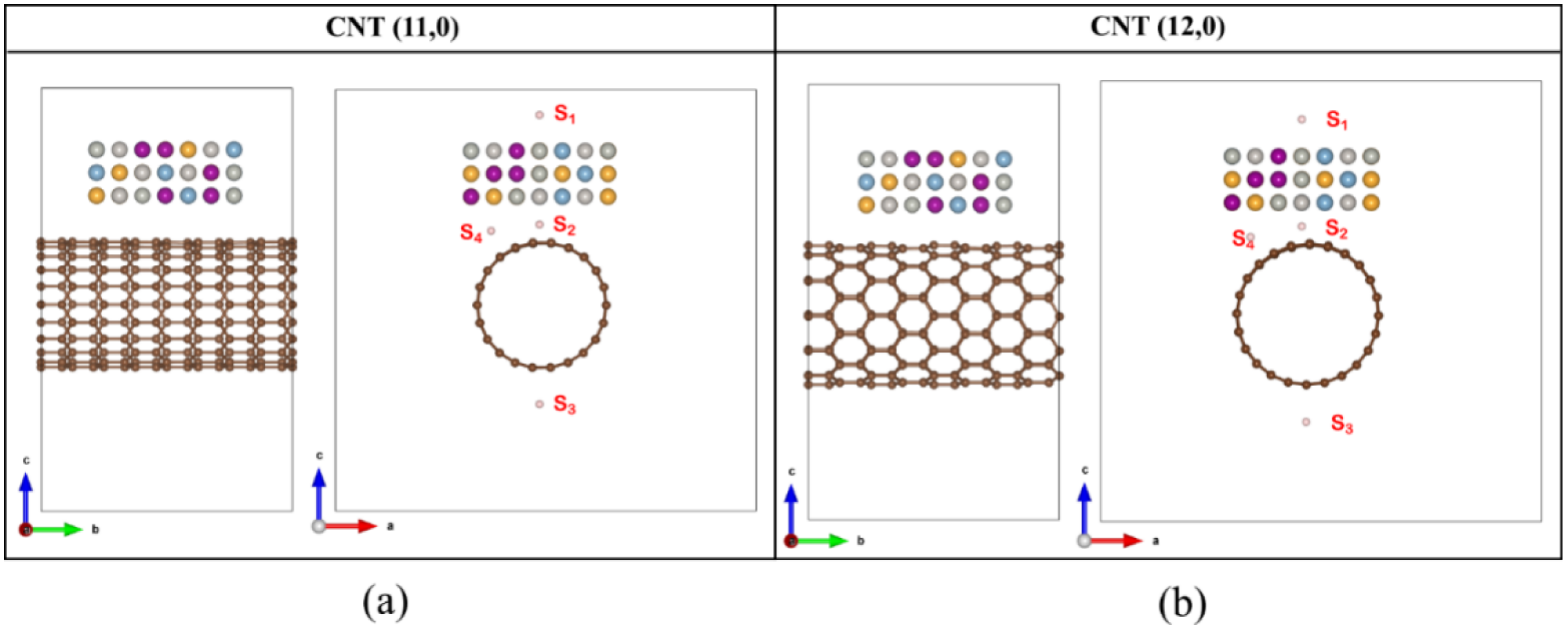
Models considered in this work. Single-walled carbon nanotubes with a semiconductor (a) and metallic (b) nature, along with a two-dimensional quasicrystal model, were examined. Four adsorption sites for the H_2_ molecule were considered, which are indicated as S_1_, S_2_, S_3_, and S_4_.

To investigate the interaction effect between the AlMnAuPdPt quasicrystal (QC) and carbon nanotubes (MWCNTs), we developed two types of models, as shown in Figure 2. These models represent a monolayer of the quasicrystal interacting with a single-walled carbon nanotube (SWCNT) of a metallic nature (12,0) and another of a semiconductor nature (11,0). This enables a more direct and clearer interpretation of how the quasicrystal affects each type of nanotube based on its metallic or semiconductor nature and allows for a straightforward description of its interaction with the H_2_ molecule. The choice of SWCNTs instead of MWCNTs simplifies the study system by reducing interference factors arising from interlayer interactions in MWCNTs. The electronic modifications observed in SWCNT simulations are directly applicable to MWCNTs, as the sensor response is primarily governed by the outermost layer.^36^ Moreover, charge transfer mechanisms and adsorption energy calculations from SWCNTs offer critical insights into the behavior of MWCNT-based sensors upon exposure to H_2_. Additionally, SWCNT simulation is computationally less demanding than MWCNT simulation, optimizing resource usage without compromising the relevance of the analysis. The quasicrystal (QC) was generated using a face-centered cubic lattice with a lattice parameter of *a* = *b* = *c* = 3.12 Å. A random distribution of atoms was used while maintaining equimolarity, resulting in a two-dimensional QC with dimensions of 9.36 × 9.36 × 3.12 Å.

Four adsorption sites for the H_2_ molecule were evaluated, as shown in Figure 2a,b. Sites S_1_ and S_3_ correspond to the direct adsorption of H_2_ on the quasicrystal and the SWCNT, respectively, while adsorption sites S_2_ and S_4_ represent the adsorption of H_2_ at the SWCNT–QC interface. The simulations were carried out by using Density Functional Theory (DFT) implemented in the SIESTA software. GGA-PBE type exchange and correlation functionals were used to describe the electron–electron interactions.^37^ To describe the electron–ion interaction, the Troullier–Martins set of norm-conserving pseudopotentials in the Kleinman–;Bylander form was used with a doubly polarized ζ basis set. The structural relaxations were performed using a real-space mesh cutoff of 350 Ry. An electronic temperature of 300 K was used to smooth out the Fermi step function. The convergence criterion for the density matrix was set at 10^–4^. The optimization of the SWCNT and QC structures was carried out using the conjugate gradient (CG) algorithm with a convergence criterion of the Hellmann–Feynman forces of 0.02 eV Å^– 1^. A block-rigid relaxation constraint was adopted for QC, while the CNT is fully relaxed. The Monkhorst–Pack^38^*k*-sampling grid set was 1 × 1 × 1. To avoid spurious interactions between periodic images of the SWCNT@QC system, an ∼14 Å buffer vacuum space was used along the *c*-axis, obtaining a total energy variation of 2 meV, indicating that this vacuum value is sufficient to reduce the interactions due to these periodic interactions.

## 3 Results and Discussion

### 3.1 Materials Characterization

**Figure 3:**
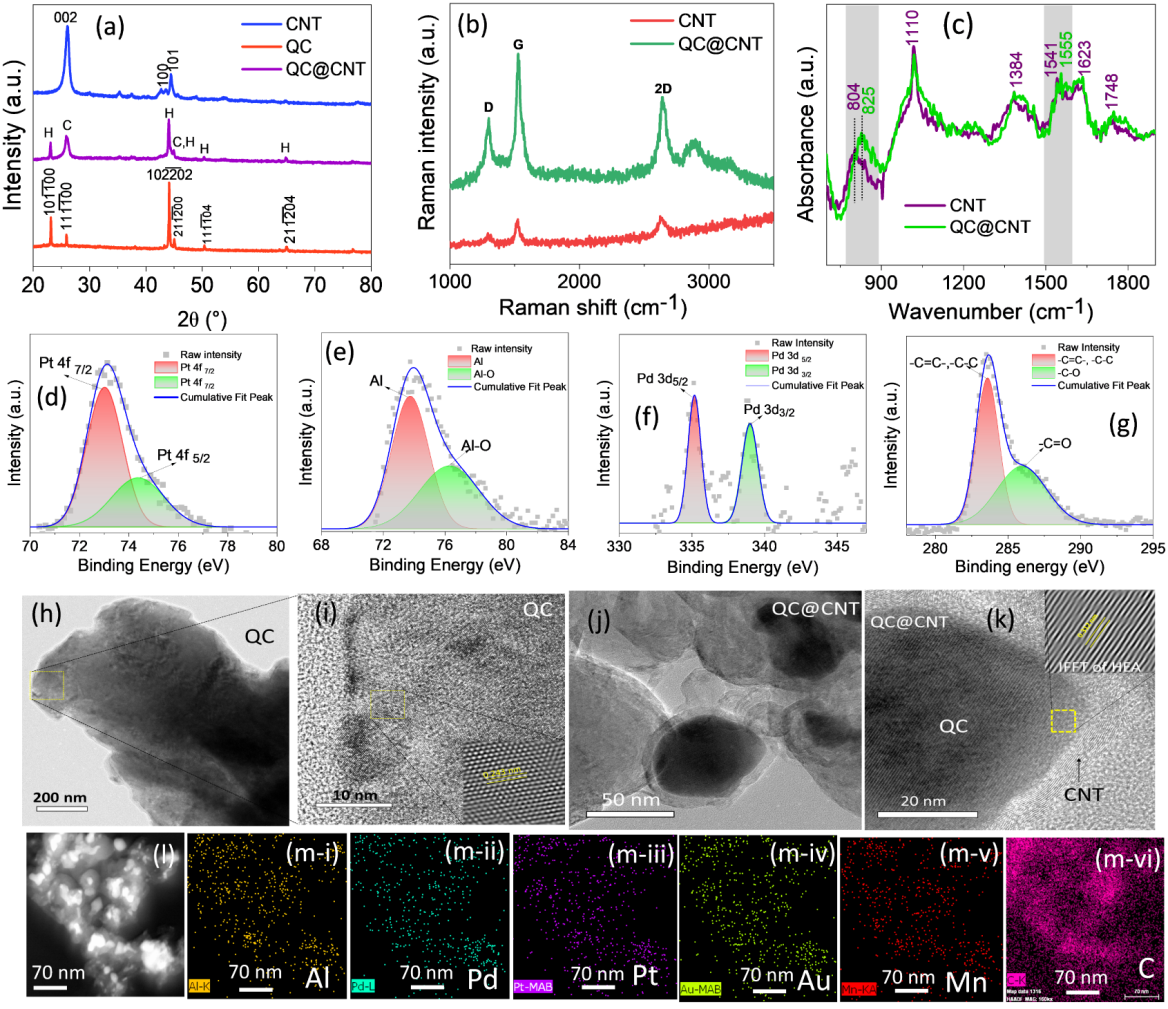
(a) XRD patterns of QC, CNT, and QC@CNTs. (b) Raman analysis of QC and QC@CNT. (c) FTIR spectra of MWCNT and QC@CNT. Elemental XPS spectra of the QC@CNT nanocomposite (d) Pt, (e) Al, (f) Pd, and (g) carbon. (h) Bright field TEM micrograph of the AlMnAuPdPt QC nanosheet. (i) HRTEM of QC with FFT-filtered HRTEM. (J) Bright field TEM micrograph of QC@CNT. (k) HRTEM interface image of QC@CNT with FFT-filtered HRTEM. Compositional elemental mapping of an AlMnAuPdPt QC@CNT. (l) High-angle annular dark-field scanning TEM (HAADF-TEM) and (m-i to m-vi) corresponding elemental distribution images.

Figure 3a depicts typical XRD patterns of the QC, MWCNT, and QC@MWCNT. The XRD pattern of QC@MWCNT perfectly matches the prominent planes of the Al-based HEA QC.^39^ The planes like (), (), (), (), and () of the HEA are quite vivid here. Additionally, the presence of two prominent planes of MWCNT^40^ (002) and (101) indicates the nanocomposite formation. Raman spectra of the pristine CVD-grown MWCNTs and the QC@MWCNT are shown in Figure 3b. Raman spectroscopy, which is the inelastic scattering of light caused by the absorption or emission of phonons, reveals valuable information on the structure and chemical bonding of MWCNTs. The D-band (1314 cm^–1^) arises by defects and sp^3^ hybridized carbon atoms, and its height is inversely associated with the quality of the nanotube (i.e., disorder in the graphitic material). The G-band (∼1524 cm^–1^) corresponds to the sp^2^-hybridized carbon atoms in the nanotube wall and accurately assesses the sample’s graphitization. The G′ or 2D peak (about 2642 cm^–1^) results from two-phonon second-order scattering and shows a long-range order in the sample. The defect density or degree of disorder in sp^2^-hybridized carbon material is determined by the ratio of the intensities of the defect or disorder-induced D-band to the symmetry-allowed graphitic band or G-band (*I*_d_/*I*_g_).^41^ The ratio *I*_2d_/*I*_g_ may be used to determine the number of walls in a particular MWCNT.^42^ It should also be observed that increasing the number of layers results in a significant decrease in the peak intensity of the 2D peak, which becomes nearly indistinguishable when the number of layers increases.^43^Figure 3c represents the Fourier transform infrared (FTIR) spectra of CNT and QC@CNT. The prominent vibrational modes of CNT at 804 cm^–1^, 1110 cm^–1^, 1384 cm^–1^, 1541 cm^–1^, and 1748 cm^–1^ wavenumbers show the C–H stretching, −OH vibration, C–O stretching, C=C/C–C stretching, and C=O stretching, respectively.^44,45^ The adsorption of the CNT onto the 2D sheets of the multicomponent alloy has been confirmed from the IR spectra of QC@CNT by the shifting of the vibrational modes toward the higher wavenumbers, from 804 cm^–1^ and 1541 cm^–1^ to 825 cm^–1^ and 1555 cm^–1^, respectively. This shows the binding of the HEA atoms to the C=C/C–C bonds of the CNT.^44^Figure 3d–g displays the high-resolution XPS spectra of the QC@CNT composite to identify the elemental composition.^46^ The deconvoluted XPS spectra of Pt 4f in Figure 3d reveal two distinct peaks at 72.5 eV and 74.6 eV, corresponding to Pt 4f_7/2_ and Pt 4f_5/2_, respectively.^47^ In Figure 3e, the deconvoluted XPS spectra of Al indicate the presence of Al metal by the peak at 73.7 eV along with the smaller oxide peak at 76 eV.^48^Figure 3f demonstrates the presence of Pd metal by the peaks at 335.2 and 339.8 eV, which corresponds to Pd 3d_5/2_ and Pd 3d_3/2_, respectively.^49^ Additionally, the presence of MWCNT along with HEA in the nanocomposite has been confirmed by the elemental spectra of C 1s in Figure 3g. It shows the elevated peak at 283.6 eV denoting the C=C and C–C bonds of the MWCNT and the lower peak at 286 eV represents the C–O bonds.^50,51^

To understand the morphology and interaction between MWCNT and AlMnAuPdPt HEA QC sheet, a detailed TEM analysis was carried out. A bright-field TEM micrograph of the AlMnAuPdPt HEA nanosheet is presented in Figure 3h showing a sheet-like morphology, and a corresponding high-resolution image from the boxed region in Figure 3i indicates the spacing between two neighboring fringes to be approximately 0.243 nm. The TEM analysis of the AlMnAuPdPt HEA QC@MWCNT composite sample is presented in Figure 3j. As is evident, Figure 3k consists of features identical to those of the HEA nanosheet and MWCNT, HEA nanosheet wrapped with MWCNT. Figure 3l displays the high-resolution TEM image of the MWCNT and HEA QC nanosheet with a magnified view of the highlighted region. From Figure 3l, it can be observed that there is no physical bonding between the HEA-QC and the MWCNTs. The high-angle annular dark-field transmission electron microscopy (HAADF-TEM) and energy-dispersive X-ray spectroscopy (EDS) were also carried out to identify the uniform distribution of Al, Mn, Au, Pd, Pt, and carbon, as shown in Figure 3m(i–vi). The primary characteristic of the QC@CNT composition material is its chemical homogeneity or uniform distribution of all elements at the microscopic length scale. This has been confirmed further by the element mapping and EDS pattern linked to the HAADF-TEM, as seen in Figure 3m(i),m(vi).

### 3.2 Gas-Sensing Characterizations

**Figure 4:**
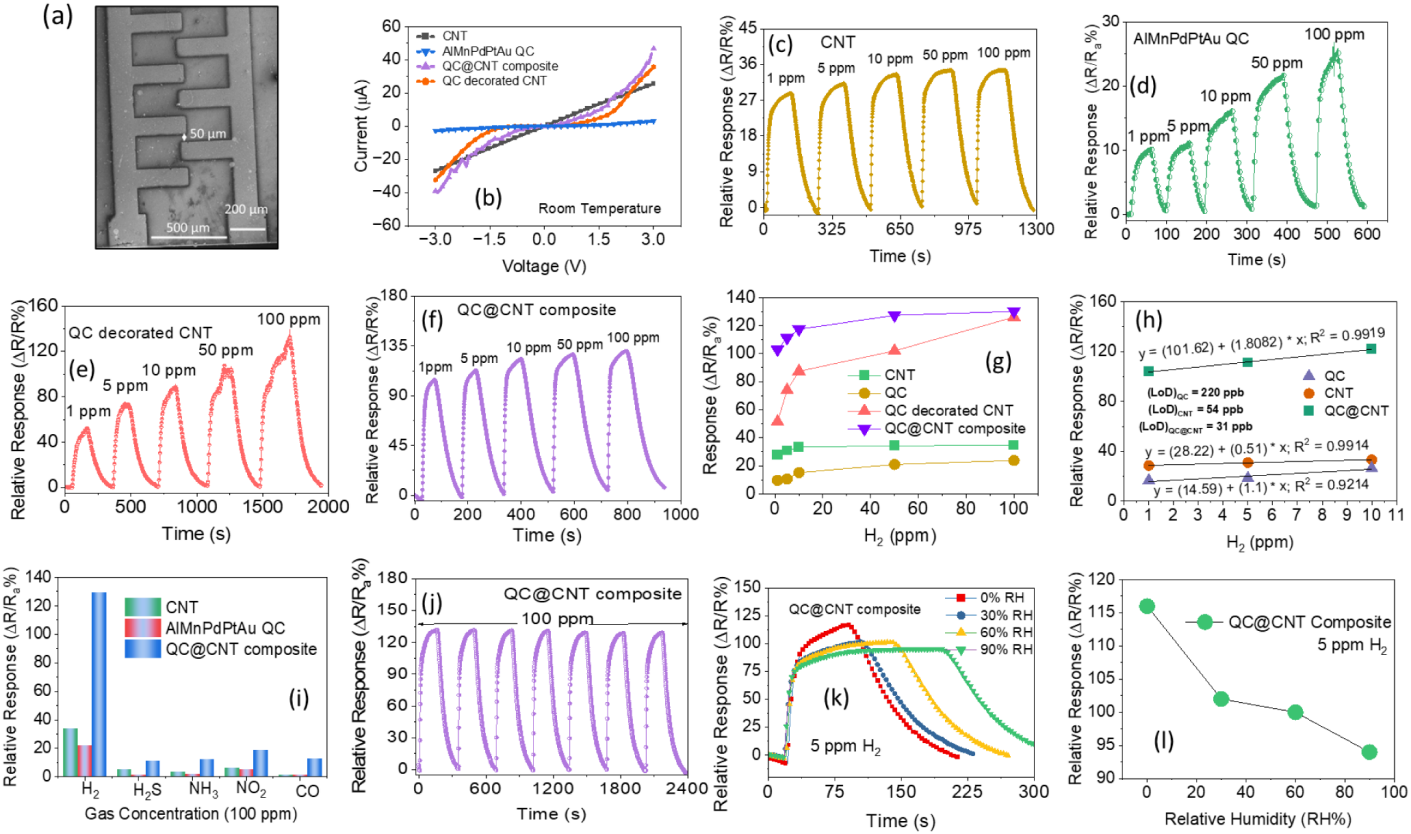
(a) SEM image of the sensor device. (b) *I*–*V* characteristics of the sensor bare CNT, bare QC, QC-decorated CNT, and QC@CNT composite at RT. (c–f) Dynamic relative response curves of sensor bare CNT, bare QC, QC-decorated CNT, and QC@CNT composite at RT. (g) Comparative graph of corresponding relative response curves (c–f). (h) Linear fitting response and LOD calculation of sensors CNT, QC, and QC@CNT composite. (i) Comparative selectivity test bar graph of sensor CNT, QC, and QC@CNT composite at RT. (j) Dynamic cyclic response curves of the QC@CNT composite sensor at 100 ppm of H_2_ to test the repeatability of the sensor. (k) Dynamic relative response curves of optimum response giving sensor QC@CNT at different RH% to 5 ppm of H_2_ at RT, and (l) comparative response curve of corresponding curves (k).

Gas-sensing tests were conducted by using a constant bias voltage of 3 V applied to two-terminal devices. The SEM image of the device is shown in Figure 4a. A Keithley Source Meter (2636B) was used to obtain a real-time resistance signal. The *I*–*V* characteristics of the sensors are shown in Figure 4b. The *I*–*V* curve of bare CNT shows a symmetric, near-linear behavior. This suggests that CNT exhibits typical semiconducting behavior, though with some linearity due to its metallic nature. At room temperature, CNTs often demonstrate moderate conductivity due to their band structure, where both metallic and semiconducting nanotubes can contribute to the current flow. AlMnPdPtAu QC nanosheets have an aperiodic but ordered structure, which significantly affects their electronic properties. In typical crystalline solids, Bloch states enable delocalized electrons to flow relatively freely. However, in quasicrystals, the lack of periodicity often leads to electron localization, where electrons become trapped in localized states, limiting their mobility. Due to the aperiodic atomic arrangement, quasicrystals tend to have low carrier mobility. This explains the relatively small current values (in μA range) despite applying up to 3 V. AlMnPdPtAu-decorated CNT indicates a more enhanced conductivity compared to the bare CNT. Decoration of CNT with the quasicrystal seems to improve charge transport properties. This could be due to the introduction of additional charge carriers or a modification in the energy band alignment between the quasicrystal and the CNT, enhancing electron flow. However, the QC@CNT composite shows a more significant current increase, particularly at higher positive and negative voltages. This suggests a synergetic effect between the QC and CNT, possibly due to enhanced charge transfer at the heterointerface. The heterostructure may reduce barriers to electron or hole injection, leading to increased current at applied voltages. Before conducting in-depth sensing experiments, a preliminary measurement was conducted on the pure CNT and pure AlMnPdPtAu QC shown in Figure 4c,d. Furthermore, the sensing effect of the optimum amount of QC nanosheet-decorated CNT and QC@CNT composite was also examined in Figure 4e,f. This, together with the reducing nature of H_2_, suggests that the sensors are consistent with the p-type nature and the resistance of sensors increases after exposure to H_2_, as shown in Figure S1a–d. The measurements were recorded at room temperature (27 ± 2 °C) and a relative humidity of 40%. The relative response is calculated as [(*R*_g_ – *R*_a_)/*R*_a_] × 100], where *R*_a_ and *R*_g_ are the sensor resistance in air and target gas, respectively. The lower adsorption energy of the H_2_ gas molecules on the sensor surface might lead to a complete recovery. Figure 4g shows the comparative response curves of the sensors for pure CNT, pure QC, the optimum amount of QC-decorated CNT, and QC@CNT composite, where the QC@CNT composite sensor response varied from 103% (1 ppm of H_2_) to 130.4% (100 ppm H_2_), which is approximately 5.4 times, 4 times, and 1.1 times larger than the bare pure AlMnPdPtAu HEA QC, CNT, and QC-decorated CNT sensor, respectively, for 100 ppm of H_2_. Composites have clear advantages over HEA QC and CNTs due to the transfer of charges through the connected pathways formed by the QC@CNT in the composite. This leads to faster resistance variation, making composites a better choice. The QC@CNT sensor exhibits a higher relative response due to factors such as its larger specific surface area, high charge carrier density, and the synergistic effects that arise when a composite is formed. Figure 4h shows the fitting curves of the bare MWCNT, QC, and QC@MWCNT composite sensor. It can be observed that the sensor response increases with an increasing H_2_ concentration until saturation. Additionally, the response of the composite sensor to low concentrations of H_2_ shows a linear relationship with H_2_ concentration (eq 1), and the straight lines of the two sensors are fitted according to the experimental data as follows:
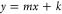
1

where *y* is the response of sensors, *m* means the sensitivity of the sensor to H_2_, *x* is the concentration of H_2_, and *k* is the intercept of the fitted line. The fitting line has good linearity, and the limit of detection (LOD) is 31 ppb. Equation 2 can be used to calculate the sensor’s LOD:
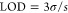
2

where σ is the standard deviation of the calibration curve and *s* is the slope of the calibration curve.^52^

Selectivity is the key index used to evaluate gas sensitivity. The response of sensors to 100 ppm of H_2_, H_2_S, NH_3_, NO_2_, and CO is compared in Figure 4i, the composite sensor is much more responsive to H_2_ than other gases. Figure S2a–c shows the transient relative response curves of the selectivity test of CNT, QC, and QC@CNT composite sensors toward various gases (H_2_, H_2_S, NH_3_, NO_2_, and CO) at RT toward 100 ppm concentration. The sensor greatly enhanced the selectivity to H_2_ compared to the pure CNT and pure QC sensor. Figure S3 shows a comparison of the response of pure H_2_ gas and a mixture of gases. The graph compares the relative response of a sensor to 5 ppm of H_2_ in pure and mixed gas environments at room temperature (RT). The curves represent the response to H_2_ alone, while the curve corresponds to a mixture of H_2_ with NH_3_, H_2_S, NO_2_, and CO. The mixed gas environment results in a higher response, indicating possible synergistic or competitive adsorption effects among the gases. The prolonged recovery suggests stronger interactions or slower desorption in a mixed environment. The repeatability test of the QC@CNT composite sensor to 100 ppm of H_2_ was tested, and as shown in Figure 4j, the sensor shows consistency in each cycle. The effect of humidity (RH, from 0% to 90%) on the sensing properties of the optimum composite sensor is also analyzed. The change in resistance (Δ*R*) decreases gradually with the increase in RH%, which is thought to be caused by excess H_2_O molecules adsorbed on the surface (Figure 4k). Temporal resistance curves with relative humidity (RH%) for 5 ppm of H_2_ at RT of the optimum sensor QC@CNT are shown in Figure S4. The H_2_O molecules occupy the active H_2_ adsorption sites, resulting in a reduced sensing response. However, even in a 90% RH environment, a reliable response to H_2_ is still 94% to 5 ppm (Figure 4I), suggesting great application potential. The response time (τ_res_) and recovery time (τ_rec_) of CNT and QC@CNT for 100 ppm of H_2_ concentration at RT have been calculated in Figure S5. Figure S6 illustrates the relative response of a QC@CNT composite sensor to 5 ppm of H_2_ at different operating temperatures. The response increases significantly with temperature, peaking around 200 °C. At lower temperatures (27 °C and 50 °C), the sensor exhibits a slower response and recovery. The highest response is observed at 200 °C, indicating enhanced reaction kinetics and adsorption–desorption dynamics at elevated temperatures. The rapid increase and subsequent decrease suggest efficient gas interaction with the composite material. Figure S7 presents the 4-week long-term stability of the QC@CNT composite sensor for detecting 1 ppm of H_2_ over 4 weeks. The sensor maintains a consistent response pattern, with distinct adsorption and desorption cycles observed each week. While slight variations in peak response are visible, the overall stability suggests reliable repeatability. The minimal drift in response highlights the sensor’s robustness for long-term hydrogen detection. Table 3 compares the present sensor performance with those of other previously reported H_2_ sensors.

## 4 Sensor Mechanism

**Figure 5:**
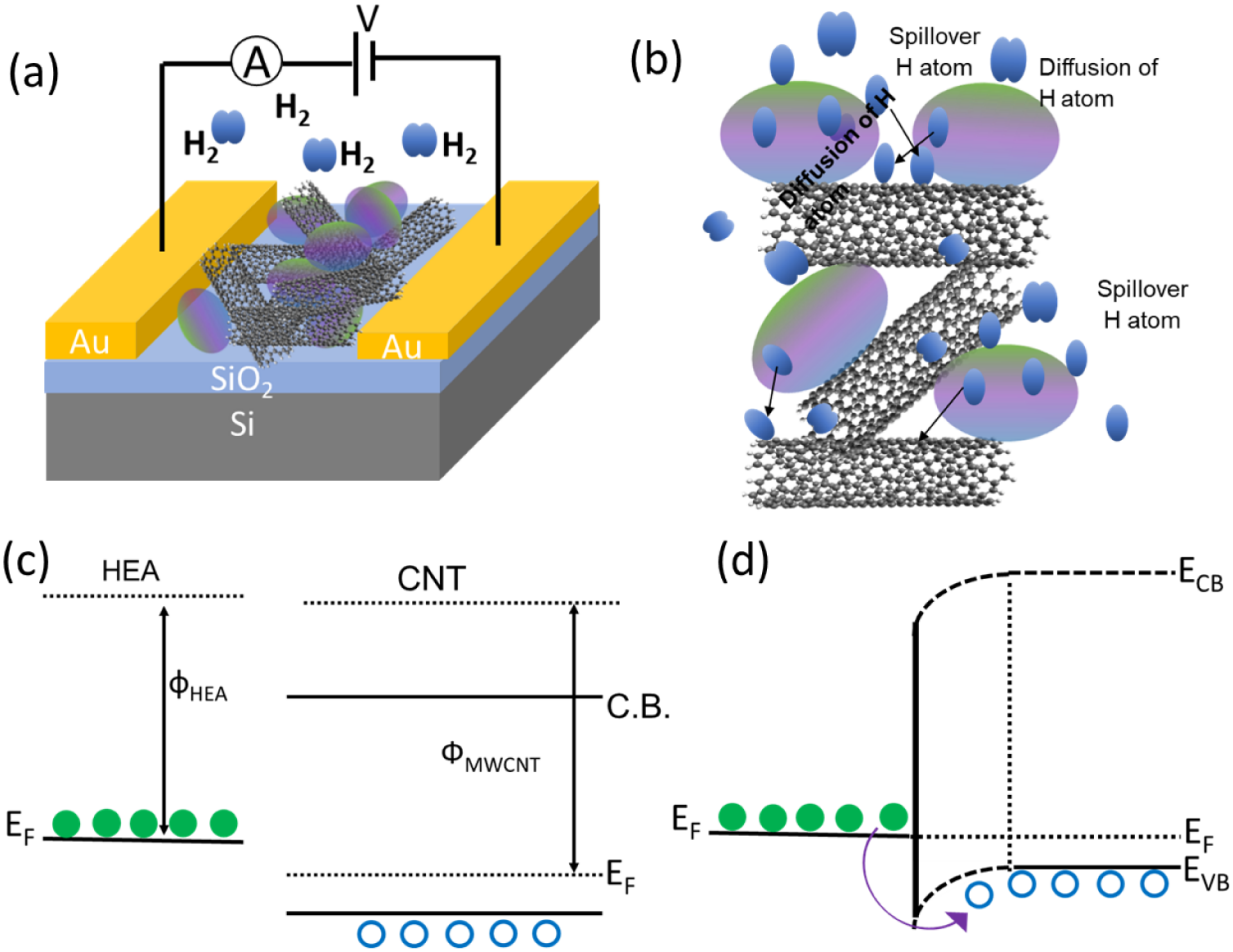
Mechanism of exploration of QC@MWCNT-based H_2_ sensors. (a) Equivalent circuit scheme of the sensor and (b) schematic of H_2_ on QC@MWCNT interaction. Band diagram of different stages during H_2_ sensing (c) before junction formation and (d) after junction formation.

The electrical properties of semiconducting CNTs are highly influenced by charges transferred from gas molecules, whereas metallic CNTs are unaffected by molecule absorption, resulting in minimal changes to the density of states near the Fermi level.^53^ Incorporating catalytically active metal atoms on the surface of carbon nanotubes, performance can be enhanced through controlling the Fermi level and spillover mechanism. Changes in electronic transport result in the control of the Fermi level due to a depletion region.^54^ The spillover model defines the increased adsorption of gaseous species on metals and their later migration onto the sensor material.^55^ Through the dissociative adsorption of hydrogen atoms^56−58^ and the subsequent transfer of adsorbed gas molecules/atoms to MWCNT, the surface adsorption for hydrogen is enhanced by noble metal elements, particularly Pd,^59^ and alloys of noble metals. Previous studies found that incorporating Au into the Pd–Pt alloy improved the sensitivity and efficiency of H_2_ gas sensing. Using the Au element enhances the response and minimizes the apparent activation energy for hydrogen sorption.^60,61^ This results in improved charge transfer, resulting in a boosted catalysis or sensor response. However, as depicted, the functionalization of HEA NPs affects the electronic levels of the semiconductor. The barrier presents at the junction between the metal and semiconductor as a result of charge transfer. Metals, known for their electron-donating properties, are pumped into carbon nanotubes. In the case of p-type semiconductors, the electrons recombine with some of the holes (Figure 5b,c), which are the majority of charge carriers. This recombination results in the restoration of the Fermi level toward the intrinsic limit. This enhances the resistance of the semiconductors. The formation of nanoscale barriers at the HEA nanoparticles and MWCNT junctions improves sensing because hydrogen chemisorption causes a significant change in resistance. The rate at which the sensor arrives at saturation and recovery is controlled by the kinetics of the surface adsorption.

## 5 DFT Computational Analysis

**Figure 6:**
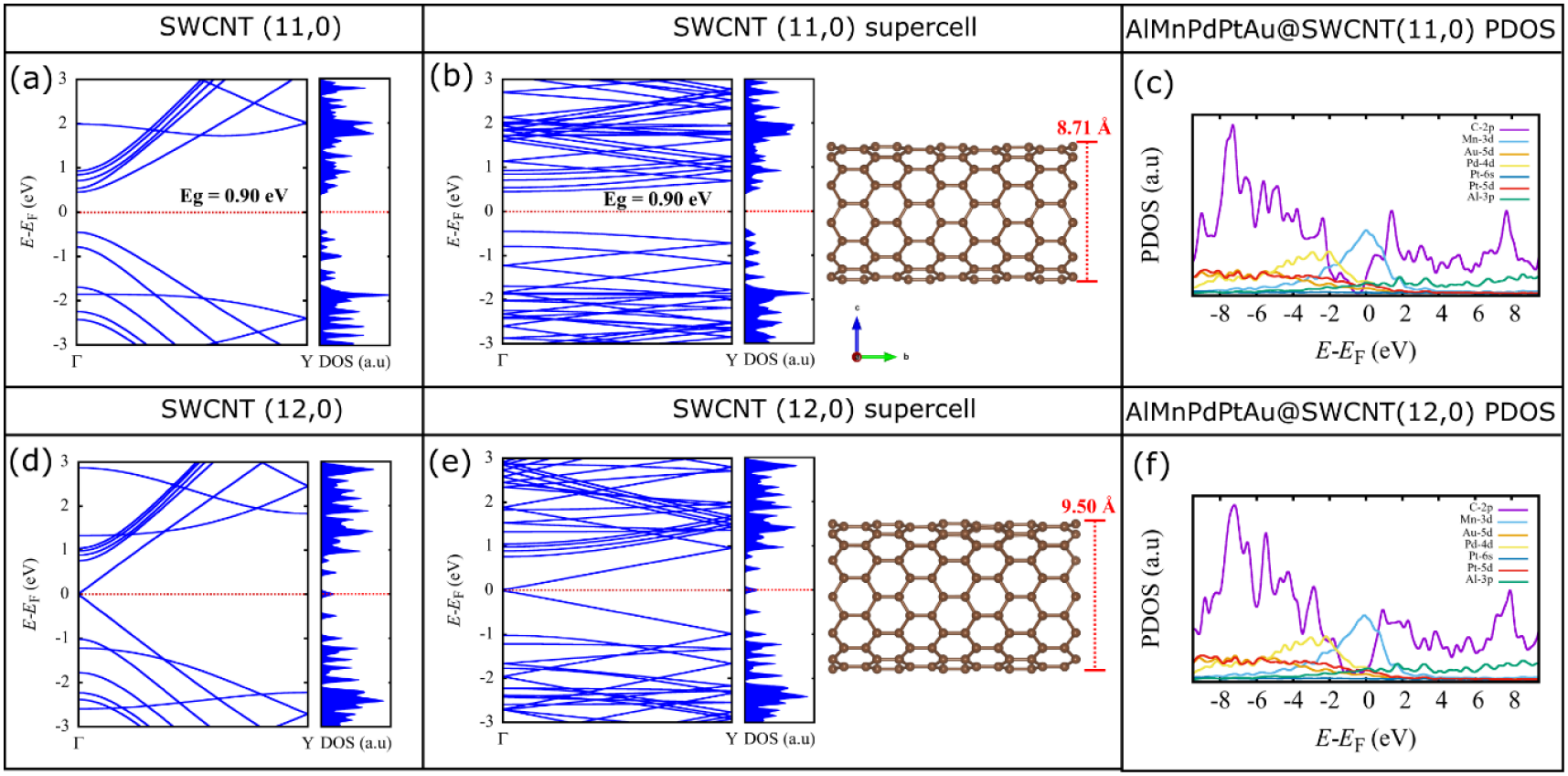
(a, b) Electronic band structure and density of the minimal unit cell of SWCNT (11,0). (c) Partial density of states (PDOS) of the AuMnPdPtAu@SWCNT (11,0) system. (d, e) Electronic band structure and density of the minimal unit cell of SWCNT (12,0). (f) Partial density of states (PDOS) of the AuMnPdPtAu@SWCNT (12,0) system.

Figure 6 shows the electronic band structure and density of states for SWCNTs (11,0) and (12,0) using both a minimal unit cell and a supercell with diameters of 8.71 and 9.50 Å, respectively. It can be observed that the semiconductor (with a band gap of 0.90 eV) and metallic nature of the SWCNTs are preserved when a supercell is used.^62^ This result is significant, as it confirms the retention of the electronic properties of the SWCNTs when employing supercells, which were subsequently used to study the interaction of the SWCNTs with the quasicrystal and the H_2_ molecule. The partial density of states (PDOS) for the QC@SWCNT (11,0) and QC@SWCNT (12,0) systems is shown in Figure 6c–f. For both systems, the contribution of Mn-3d orbital states is predominant near the Fermi level. This suggests that the interaction of H_2_ with the QC@SWCNT system may be primarily localized on the Mn atoms within the quasicrystal.^63^

**Figure 7:**
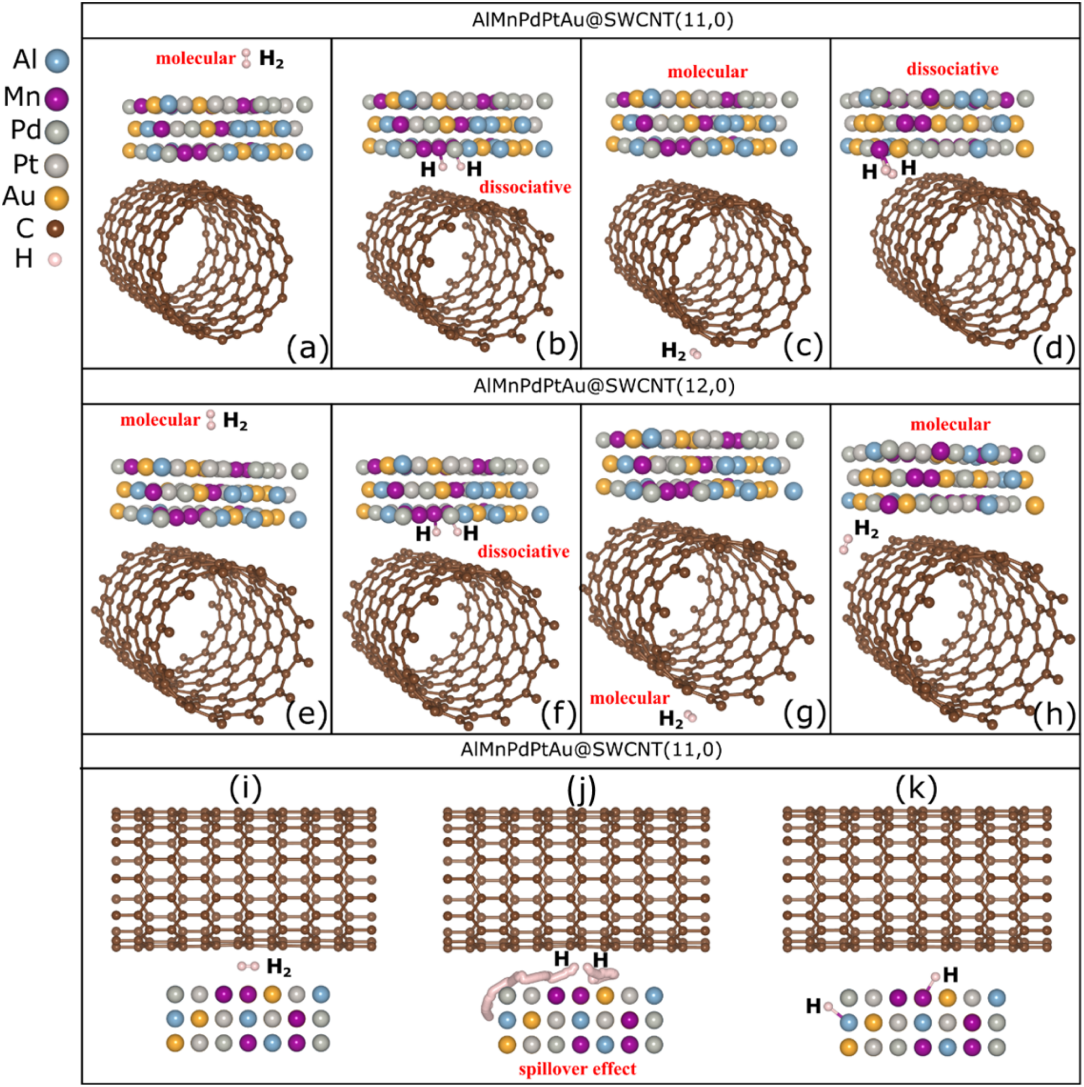
Optimized final configurations of H_2_ adsorption at the four adsorption sites, top (a–d) and bottom (e–h), correspond to the AlMnPdPtAu@SWCNT (11,0) and AlMnPdPtAu@SWCNT (12,0) models, respectively. Each panel shows the type of H_2_ adsorption (molecular or dissociative) on the AlMnPdPtAu@SWCNT system. Adsorption process of the H_2_ molecule on the AlMnPdPtAu@SWCNT (11,0) system, with the AlMnPdPtAu QC positioned at the bottom. Panels (i) and (k) show the initial and final configurations, respectively, while panel (j) displays the trajectory of H atoms as they spill over onto the AlMnPdPtAu structure.

The optimized structures for H_2_ molecule adsorption at the four adsorption sites described in Figure 2 are shown in Figure 7 for both the semiconductor QC@SWCNT (11,0) and metallic QC@SWCNT (12,0) systems. As seen in Figure 7a,c, which corresponds to adsorption sites S_1_ and S_3_ of the structure of QC@CNT (11,0), the H_2_ adsorption modes are molecular type, where the H_2_ molecule geometry is preserved and no chemical bonds are formed. In contrast, Figure 7b,d, representing the adsorption sites S_2_ and S_4_, which correspond to adsorption sites at the QC@SWCNT interface, shows a dissociative adsorption mode in which the H_2_ molecule is dissociated and forms chemical bonds with manganese atoms, with an Mn–H bond distance of approximately 1.68 Å. Adsorption modes similar to those are also observed in the QC@SWCNT (12,0) system, as shown in Figure 7e,f. This demonstrates that H_2_ adsorption is independent of the semiconductor or metallic nature of the SWCNTs.

The adsorption of H_2_ at the interface of the QC@SWCNT (11,0) system, with the quasicrystal positioned at the bottom, is illustrated in Figure 7i–k. Figure 7i,j depicts the initial and final configurations of H_2_ adsorption, respectively, while Figure 7k shows the trajectory of the dissociated hydrogen atoms from the H_2_ molecule. As observed, the H atoms migrate across the QC surface until stabilizing and bonding with Mn atoms. The displacement of H atoms at the interface between the QC and SWCNT can be interpreted as a spillover effect associated with strong H_2_ adsorption and subsequent migration toward the sensor material.

**Figure 8:**
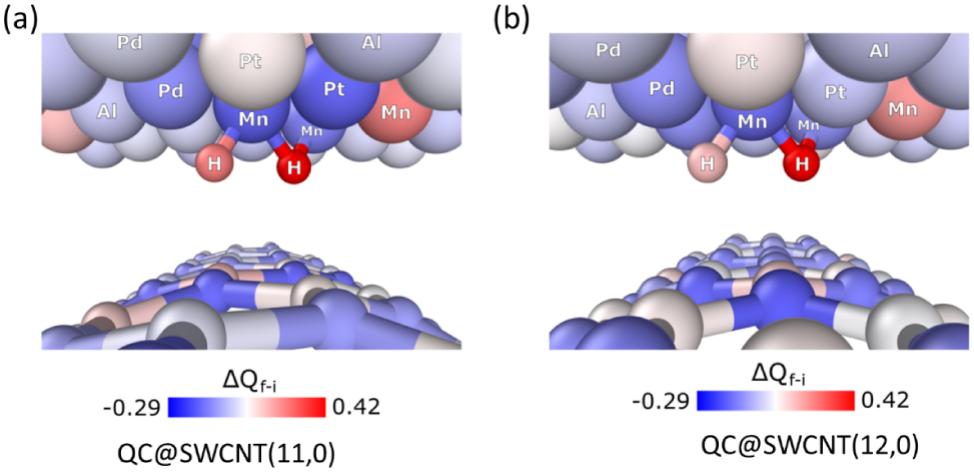
Bader charge for the S_2_ adsorption site (a) QC@SWCNT (11,0) and (b) QC@SWCNT (11,0).

The adsorption energy values at the interface exhibit significantly large negative values, indicating a dissociative adsorption mode. Furthermore, Bader analysis reveals charge transfer from the carbon nanotube to the quasicrystal. The adsorption of the H_2_ molecule on QC@SWCNT (11,0) and QC@SWCNT (12,0) has been tested on four sites: S_1_, S_2_, S_3_, and S_4_. The computed adsorption energy of the H_2_ molecule is shown in Table 2. The charge accumulation on H_2_ is further verified by Bader charge analysis. Figure 8a,b shows the Bader charge difference (Δ*Q*_f–i_) for QC@SWCNT (11,0) and QC@SWCNT (12,0) before adsorption and after absorption of H_2_ molecules. These analyses provide a detailed quantification of charge transfer, revealing a net electron donation from the quasicrystal to both the SWCNT and the hydrogen at the interface. This finding further reinforces the well-established role of transition metals with partially filled d-orbitals in governing charge redistribution (Table 3).

**Table 2:** Adsorption Energy Values of QC@SWCNT (11,0) and QC@SWCNT (12,0)

Site of Adsorption	Adsorption Energy (eV) for QC@SWCNT (11,0)	Adsorption Energy (eV) for QC@SWCNT (12,0)
S_1_	–0.70	0.08
S_2_	–5.21	–5.53
S_3_	0.38	0.12
S_4_	–1.55	0.11

**Table 3:** Comparisons of H_2_ at RT Gas Sensor-Based MWCNT Materials

Sensor Type	Gas Concentration (ppm)	Response (%)	Operating Temperature	Refs.
SWCNT and Pd	1000	75	RT	(64)
SWCNT and Pd	40 000	9	RT	(65)
MWCNT and Pd	2100	15	RT	(66)
SWCNTand Pd	400	36	RT	(30)
MWCNT	20	28	RT	(67)
CNT and WO_3_	5000	99	350 °C	(68)
SWCNT Rope and Pd	1000	78	RT	(69)
MWCNTs and Pt	10 000	3.2	RT	(70)
MWCNT/SnO_2_	1000	57.6	200 °C	(71)
Pd/SWCNT	10 000	285	RT	(72)
MWCNT	100	34	RT	this work
AlMnAuPdPt QC/MWCNT	100	130.4	RT	this work

## 6 Conclusion

To summarize, we present a strategy for high-performance hydrogen sensing using AlMnAuPdPt HEA QC nanosheets integrated with a CNT. The QC nanosheets facilitate H_2_ dissociation and adsorption through electronic charge redistribution, forming a Schottky barrier with the CNTs. This interface forms a Schottky barrier, amplifying resistance changes due to charge transfer. The resulting sensor achieves a remarkable resistance change of 4 orders of magnitude at room temperature for 100 ppm of H_2_, with a fast response time of 19 s and a detection limit as low as 31 ppb. These metrics represent the highest sensitivity reported for pure CNT-based H_2_ sensors at room temperature. Calculations performed using density functional theory reveal two distinct adsorption modes: a molecular mode, where no chemical bonds are formed, and a dissociative mode, in which H_2_ splits and forms bonds with Mn atoms in the quasicrystals. Furthermore, the spillover effect was observed, facilitating the migration and stabilization of hydrogen atoms on the quasicrystal surface. This work highlights the synergistic role of the HEA-QC and CNT heterointerface, setting a new benchmark for room-temperature hydrogen sensing.

## Supporting Information Available

Transient relative response curves of the selectivity test of sensor CNT, QC, and QC@CNT composite with various gases (H_2_, H_2_S, NH_3_, NO_2_, and CO) at RT toward 100 ppm concentration, temporal response curves with relative humidity (RH%) for 5 ppm of H_2_ at RT of optimum sensor QC@CNT, and response and recovery time comparison of sensors CNT and QC@MWCNT at RT (PDF)
