# Supplementary material for: AlMnPdPtAu Quasicrystal Modulated Carbon Nanotubes for H2 Sensors: Experimental and DFT Computational Analysis
Source: ACS Appl Mater Interfaces. 2025 Apr 1;17(14):21670–81. doi: 10.1021/acsami.5c00924 (PMC11986908; doi:10.1021/acsami.5c00924)
Supplement: Supplementary file 1 — am5c00924_si_001.pdf [file am5c00924_si_001.pdf]

Supporting Information

AlMnPdPtAu Quasicrystal Modulated Carbon Nanotubes for H_2 Sensors: Experimental and DFT Computational Analysis

Sumit Kumar[a], Anyesha Chakraborty[b], Juan Rafael Gomez Quispe[c], Rahul Mitra[d], Suraj Barala[e], Pedro Alves Da Silva Autreto[c], Chandra Sekhar Tiwary[b,d], Krishanu Biswas[d*], Mahesh Kumar[f,a*]*

[a]Department of Electrical Engineering, Indian Institute of Technology Jodhpur, Jodhpur, 342030, India

[b]School of Nano Science and Technology, Indian Institute of Technology, Kharagpur, West Bengal-721302, India

[c]Center for Natural and Human Sciences, Federal University of ABC, Santo André, SP, Brazil

[d]Department of Materials Science and Engineering, Indian Institute of Technology Kanpur, Kanpur, 208016, India

[d]Department of Metallurgical and Materials Engineering, Indian Institute of Technology Kharagpur, Kharagpur, 721302, India

[e]Inter-disciplinary Department of Space Science and Technology, Indian Institute of Technology Jodhpur, Jodhpur, India-342030

[f]Department of Cybernetics, Nanotechnology and Data Processing, Faculty of Automatic Control, Electronics and Computer Science, Silesian University of Technology, Akademicka 16, 44-100 Gliwice, Poland

Email ID- *chandra.tiwary@metal.iitkgp.ac.in, kbiswas@iitk.ac.in, mkumar@iitj.ac.in*

Supporting contents:

Figure. S1 (a–d) dynamic resistance curves of the sensors CNT, QC, QC decorated CNT, and QC@CNT composite toward (1–100 ppm) H_2 at RT.

Figure S2. (a-c) transient relative response curves of selectivity test of sensor CNT, QC, and QC@CNT composite various gases (H_2, H_2S, NH_3, NO_2 and CO) at RT toward 100 ppm concentration.

Figure S3. comparison of response of pure H_2 gas and mixture of gases

Figure S4. Temporal response curves with relative humidity (RH%) for 5 ppm of H_2 at RT of optimum sensor QC@CNT.

Figure S5. response and recovery time comparition of sensors CNT and QC@MWCNT at RT.

Figure S6. Temperature dependence H_2 sensing response on QC@CNT composite sensor

Figure S7. Four-week stability test measurements on QC@CNT composite sensor at RT for 1 ppm H_2

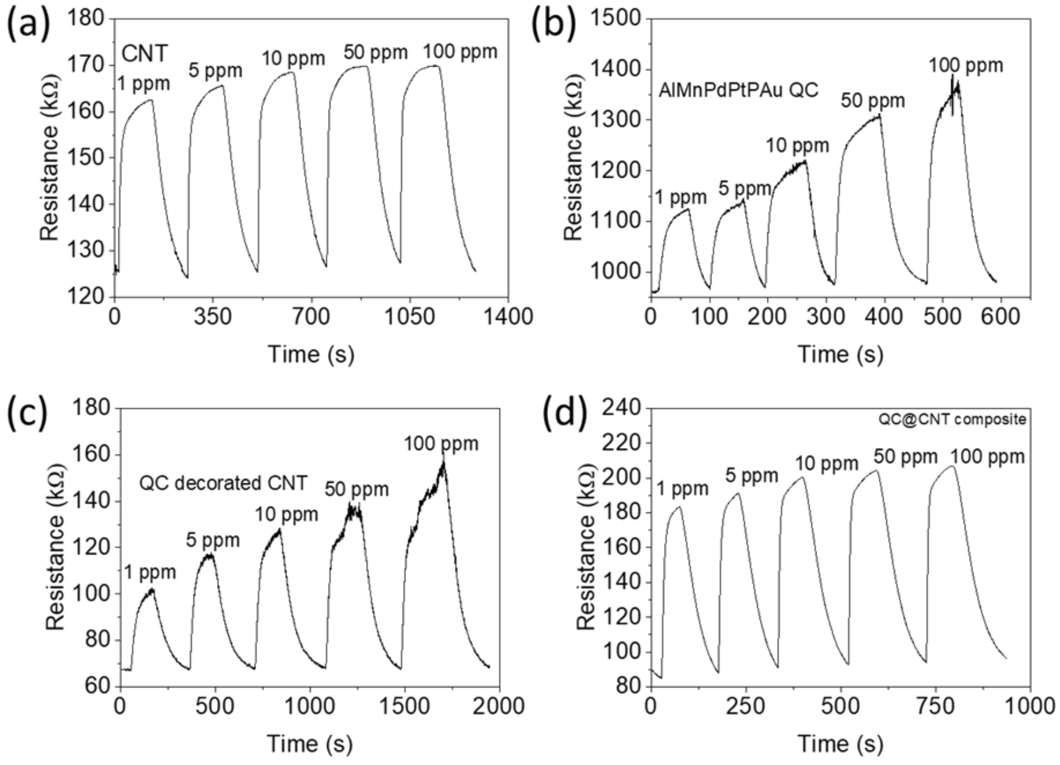

Figure. S1 (a–d) dynamic resistance curves of the sensors CNT, QC, QC decorated CNT, and QC@CNT composite toward (1–100 ppm) H_2 at RT.

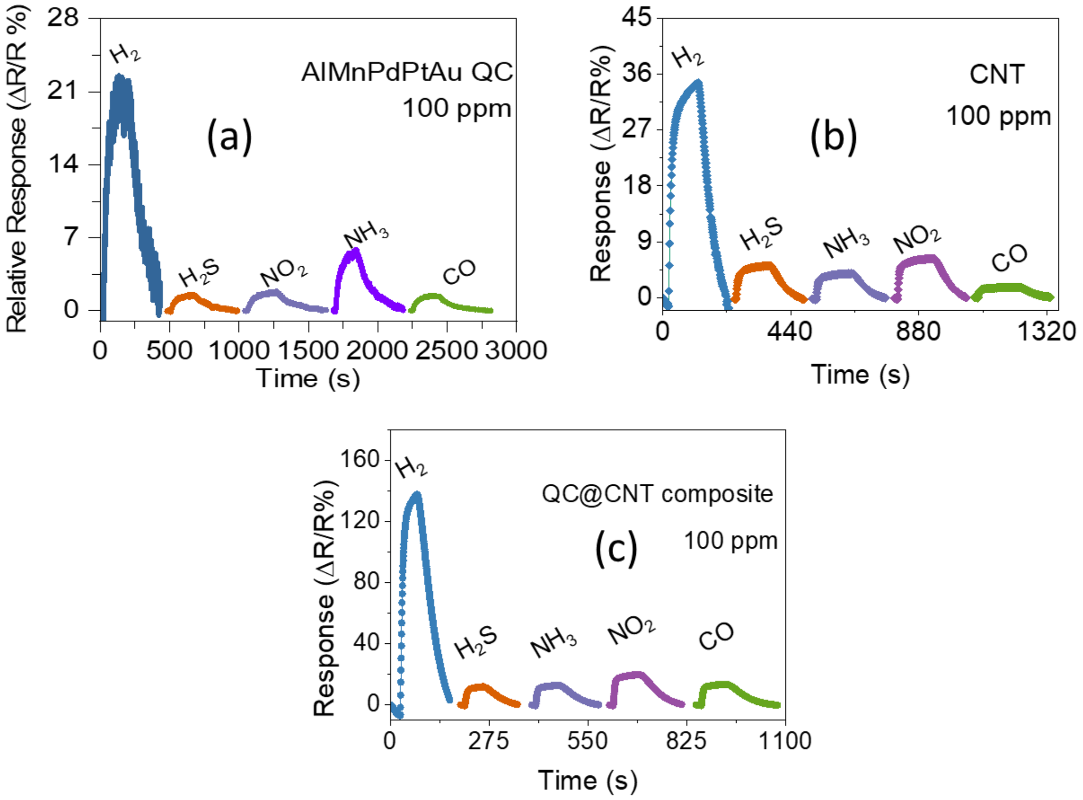

Figure S2. (a-c) transient relative response curves of selectivity test of sensor CNT, QC, and QC@CNT composite various gases (H_2, H_2S, NH_3, NO_2 and CO) at RT toward 100 ppm concentration.

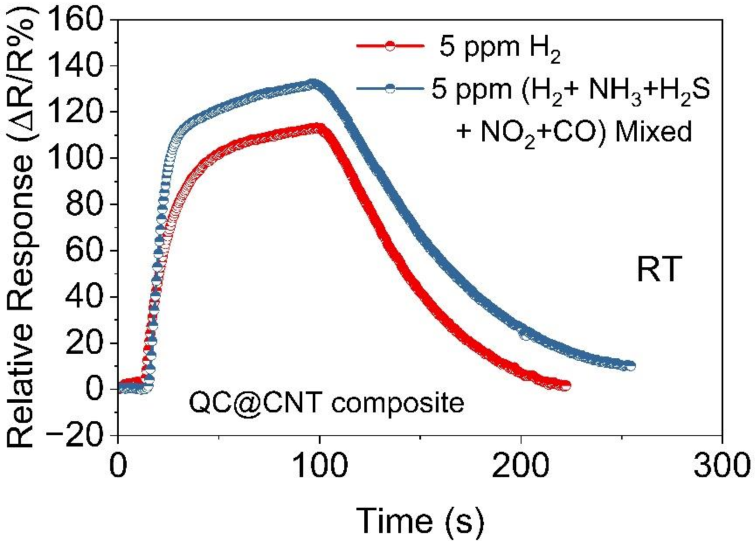

Figure S3. comparison of response of pure H_2 gas and mixture of gases.

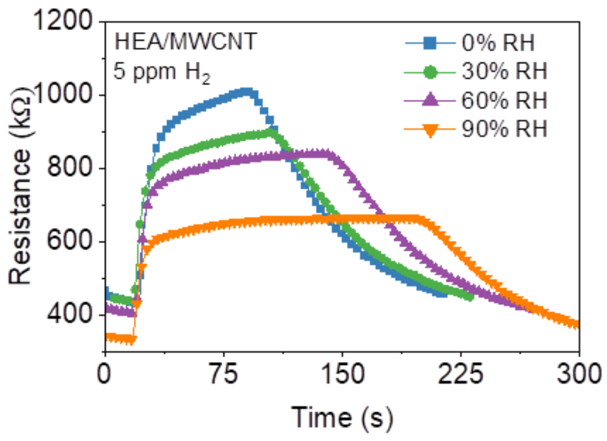

Figure S4. Temporal resistance curves with relative humidity (RH%) for 5 ppm of H_2 at RT of optimum sensor QC@CNT.

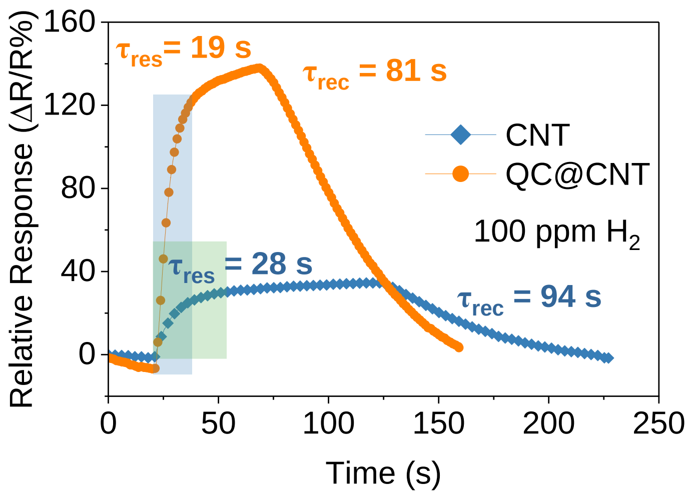

Figure S5. response and recovery time comparition of sensors CNT and QC@MWCNT at RT.

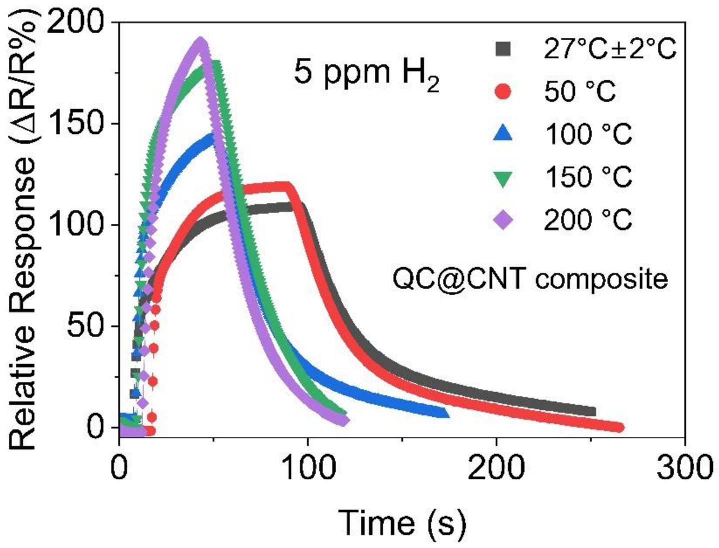

Figure S6. Temperature dependence H_2 sensing response on QC@CNT composite sensor

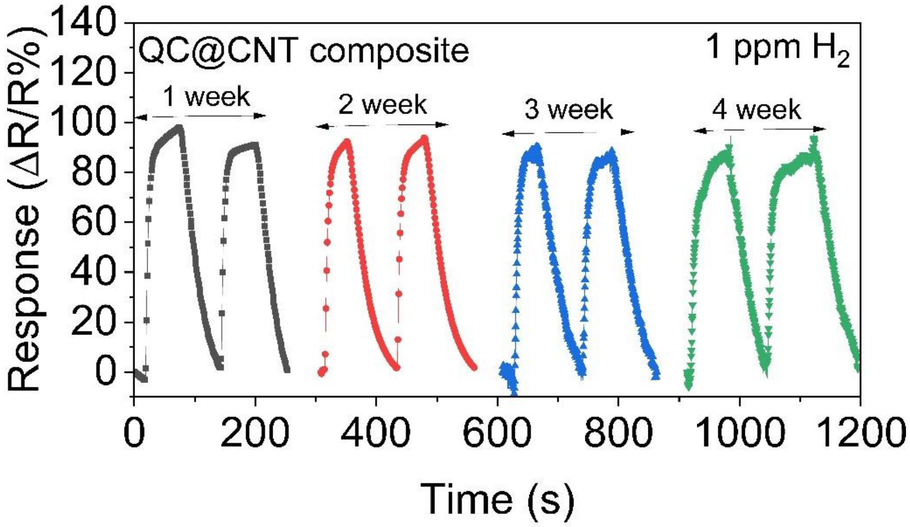

Figure S7. Four-week stability test measurements on QC@CNT composite sensor at RT for 1 ppm H_2
